# Cyclical strain modulates metalloprotease and matrix gene expression in human tenocytes via activation of TGFβ^☆^

**DOI:** 10.1016/j.bbamcr.2013.06.019

**Published:** 2013-12

**Authors:** Eleanor R. Jones, Gavin C. Jones, Kirsten Legerlotz, Graham P. Riley

**Affiliations:** Soft Tissue Research Group, School of Biological Sciences, University of East Anglia, Norwich Research Park, Norwich, UK

**Keywords:** Strain, Mechanotransduction, Transforming Growth Factor β, Metalloproteinase, Tendon

## Abstract

Tendinopathies are a range of diseases characterised by degeneration and chronic tendon pain and represent a significant cause of morbidity. Relatively little is known about the underlying mechanisms; however onset is often associated with physical activity. A number of molecular changes have been documented in tendinopathy such as a decrease in overall collagen content, increased extracellular matrix turnover and protease activity. Metalloproteinases are involved in the homeostasis of the extracellular matrix and expression is regulated by mechanical strain. The aims of this study were to determine the effects of strain upon matrix turnover by measuring metalloproteinase and matrix gene expression and to elucidate the mechanism of action. Primary Human Achilles tenocytes were seeded in type I rat tail collagen gels in a Flexcell™ tissue train system and subjected to 5% cyclic uniaxial strain at 1 Hz for 48 h. TGFβ1 and TGFβRI inhibitor were added to selected cultures. RNA was measured using qRT-PCR and TGFβ protein levels were determined using a cell based luciferase assay. We observed that mechanical strain regulated the mRNA levels of multiple protease and matrix genes anabolically, and this regulation mirrored that seen with TGFβ stimulation alone. We have also demonstrated that the inhibition of the TGFβ signalling pathway abrogated the strain induced changes in mRNA and that TGFβ activation, rather than gene expression, was increased with mechanical strain. We concluded that TGFβ activation plays an important role in mechanotransduction. Targeting this pathway may have its place in the treatment of tendinopathy.

## Highlights

• Mechanical strain regulates multiple protease and matrix genes at the mRNA level.
• Changes in mRNA level are analogous to those induced by TGFβ stimulation.
• The inhibition of the TGFβ signalling pathway abrogated the strain-induced changes.
• A SMAD activatory soluble factor is increased in activity in response to mechanical load.

## 1 Introduction

Tendinopathies are a range of diseases which are characterised by chronic tendon pain, swelling, localised tenderness, impaired movement, rupture and insidious degeneration of the tendon ECM [1–3]. They are a significant cause of morbidity and represent a sizable proportion of referrals to general practitioners [4,5]. Relatively little is known about the underlying mechanisms; however onset is often associated with an increase in age and physical activity [1,4]. Tendons predominantly affected are those exposed to higher levels of mechanical strain such as the supraspinatus, Achilles, patella and posterior tibialis tendon [3,6]. Although prevalent in the ageing general population, younger athletes also commonly affected. This is illustrated by the high incidence of Achilles tendinopathy runners [1,6]. However, due to our inadequate understanding of the disease pathology, treatment is restricted to pain relief, exercise, cryotherapy, non-steroidal ant-inflammatory drugs and surgery [1,3], all of which are often ineffective [1,7,8]. Even after corrective surgery only 60–85% of patients are able to return to sporting exercise [6]. In order to develop effective treatments we must first understand the aetiopathology of the disease.

A number of cell and molecular changes have been documented in tendinopathy such as: a decrease in type I and overall collagen content and an increase in collagen type III [9,10], increased levels of proteoglycans [11–15], increased ECM turnover and protease activity [16] and changes in cellularity [17]. An increase in Transforming Growth Factor β (TGFβ) protein has also been reported [18,19]. TGFβ is a cytokine that (in humans) exists in three isoforms (TGFβ1, TGFβ2 and TGFβ3), which are involved in many cell processes including cell proliferation, differentiation and apoptosis [20]. Newly synthesised TGFβ protein contains a propeptide known as the latency associated peptide (LAP). In the trans-golgi network, post-translational modifications involve cleavage of LAP by proprotein convertase furin [21]. TGFβ and LAP remain non-covalently bound rendering TGFβ inactive [22]. However this inhibition of TGFβ by LAP is not yet fully understood. Latent TGFβ binding proteins (LTBP) form cysteine disulphide linkages with TGFβ homodimers via LAP, to form the Large Latent Complex (LLC) [23,24]. LTBP is important in the secretion [25] and targeting of LLC to the extracellular matrix where transglutaminases covalently link the LTBP to ECM proteins such as fibronectin [26–28]. Therefore TGFβ is regulated both temporally and spatially, due to the requirement for activation and sequestration in the ECM.

In the ECM, as part of the LLC, TGFβ is a store of potentially active TGFβ. Activation has been reported to occur by a number of mechanisms: Cell surface integrins bind LAP via the RGD motif allowing protease enzymes to release active TGFβ [29]. Integrin interaction with LAP can also result in the release of active TGFβ through tensional forces between the cell and ECM [30]. The Mannose-6-phosphate/Insulin like growth factor type II receptor (M6P/IGF-II) can bind to LAP which causes cell surface localisation of the latent complex and results in activation of TGFβ through proteolytic cleavage [31,32]. Thrombospondin can interact with LAP via an LSKL motif resulting in a conformational change in the latent complex and TGFβ activation (exclusive of release from the complex) [33]. TGFβ is regulated on many levels, through intracellular post-translational modification, localisation to the ECM and through a variety of activation mechanisms.

In the canonical signalling pathway, active TGFβ binds cell surface TGFβ type I and type II receptors (TGFβRI and TGFβRII)—these receptors have serine/threonine kinase activity. Upon ligand binding, both type I and type II receptors form heterotetrameric complexes, TGFβRII phosphorylates TGFβRI, leading to phosphorylation and subsequent activation of SMAD proteins which translocate to the nucleus and cause transcriptional regulation of target genes (for review see; [20]). Since TGFβ is known to play a major role in wound healing and fibrosis, knowledge of its role and regulation in tendon is likely to be important in understanding the cause and progression of tendinopathy.

Tendinopathies are thought to be preceded by changes in cellular activity resulting in modified ECM composition, which weaken the tendon [16]; however these changes are not fully understood. Metalloproteinases are proteases which largely control the homeostasis of the ECM [34,35]. mRNA expression analysis has shown that a number of these molecules are regulated in tendinopathies [36–40]. Metalloproteinases are subdivided into a number of protein families; matrix metalloproteinases (MMPs), A Disintegrin and Metalloproteinase domain (ADAM) and A Disintegrin and Metalloproteinase domain with Thrombospondin repeats (ADAM-TS). MMPs, ADAMs and ADAM-TSs are important in the process of extracellular matrix degradation. Generally, members of the MMP family (MMP-1, -2, -8, -13 -14) mediate fibrillar (types I, II and III) collagen degradation, members of the ADAM-TS family (ADAMTS-1, -4, -5, -9, -15) degrade ECM proteoglycans such as aggrecan and versican and ADAMs are involved in processing of membrane proteins through shedding of the ectodomain [41]. Tissue inhibitors of metalloproteinases (TIMPs), of which there are four members, inhibit MMP and ADAM activity, thereby inhibiting the degradation of the ECM (for metalloproteinase reviews see: [34,42,43]).

Mechanical strain has been implicated in the development of tendinopathy, either initiating matrix damage directly or by modifying the cellular activity, although certain levels of strain are vital for the homeostasis of tendon [44–46]. Mechanical strain has been reported to modulate the expression and activity of a number of metalloproteinases [47–51], although to date no studies have investigated mechanical regulation of the entire metalloproteinase family. We have previously shown that almost all MMP and ADAMTS enzymes are expressed in normal tendon, with different patterns of expression in normal, painful and ruptured tendon [36]. Although the pattern of metalloproteinase expression was similar in Achilles and posterior tibialis tendinopathy [36,52], there are differences in the expression of some genes that we speculate may be related to differences in mechanical loading [53].

The primary aim of this study was to investigate the response of the majority of metalloproteinases and selected matrix genes to 5% cyclic mechanical strain due to the lack of existing studies on the mechanical regulation of such a wide range of genes. We hypothesised that 5% strain would give rise to anabolic changes in MMP and matrix genes. Our observations confirmed that this was the case. Secondly, since TGFβ protein expression is stimulated in tendinopathy [18,19] and pathway analysis following global gene expression profiling of chronic tendinopathic tendon tissue also showed an increase in genes related to TGFβ signalling, we were interested in the regulation of tenocyte gene expression by TGFβ. We hypothesised that strain and TGFβ would have similar affects upon metalloproteinase and matrix gene expression, due to the anabolic responses reported in both strain (in the current study) and TGFβ (in previous studies: [54–63]). The analogous response of the majority of genes to strain and TGFβ suggested that TGFβ signalling may be involved in strain regulation of gene expression. To test this hypothesis an inhibitor of TGFβRI which inhibits downstream phosphorylation of SMAD [64] was added to tenocyte cultures. We demonstrated that inhibition of TGFβRI abrogated the strain mediated response. In order to test whether TGFβ protein levels were modified in response to mechanical loading, we measured total and active TGFβ in strain conditioned medium using a cell based luciferase assay [65]. We hypothesised that TGFβ would be regulated at the protein level with mechanical loading. Our observations showed that TGFβ activation, rather than gene expression, plays an important role in mechanotransduction.

## 2 Materials and methods

### 2.1 Cell culture

Human Achilles tenocytes derived from tendinopathic tissue by explant outgrowth (< passage 10) were grown to ~ 90% confluence in Dulbecco's modified Eagle's medium (DMEM) (low glucose, glutaMAX and pyruvate [Life Technologies, Paisley, UK]) containing heat inactivated foetal bovine serum and penicillin (10 mg/ml)/streptomycin (10 U/ml) (Life Technologies, Paisley, UK) at a ratio of 100:10:1. Tenocyte seeded 3D collagen gels were prepared essentially as described by Albert Banes and colleagues [66]. In brief, rat tail collagen (2.2 mg/ml, First Link, Birmingham, UK) was mixed with  10× DMEM (Life Technologies, Paisley, UK) at a ratio of 9:1 and the pH was adjusted to approximately 7 by the addition of 10 M NaOH. Neutralised collagen and tenocyte suspension were mixed 1:1 (1 mg/ml collagen, 1.5 × 10^6^ cells/ml final density) and 200 μl was pippetted into flexible bottomed collagen I coated flexcell tissue train plates, under 20% vacuum. The cell density was selected based on pilot experiments, which showed similar changes at low and high cell densities, but the response was greater at high cell density. Gels were allowed to polymerise for 1 h at 37 °C, at which point 3 ml serum free DMEM was added. After a further 72 h medium was replaced, appropriate treatments added [TGFβ 1–5 ng/ml (R&D systems, Abington, UK), TGFβ receptor I inhibitor (SB431542) 10 μM, MMP inhibitor (GM6001) (Millipore, Watford, UK) 10 μM, serine protease inhibitor (Pefabloc) 0.5 mM, RGD peptide inhibitor and control peptide (GRGDSP and GRGESP) (Cambridge Bioscience, Cambridge, UK) [67] 7 μM, thrombospondin peptide inhibitor and control peptide (LSKL and SLLK) (Cambridge Bioscience, Cambridge, UK) [68,69] 13 mM, Mannose-6-phosphate (M6P) 10 μM and appropriate vector controls (sourced from Sigma Aldrich, Dorset, UK, unless otherwise stated)] and a uniaxal strain applied using the Flexcell FX-4000™ tissue train unit (Dunn Labortechnik, Asbach, Germany); uniaxial strain was applied in sinusoidal wave form at 5% cyclic strain at 1 Hz for up to 48 h.

### 2.2 RNA extraction and reverse transcription

Tenocyte seeded collagen gels were dissolved in  4× the gel volume of Trizol reagent (Life Technologies, Paisley, UK). RNA was isolated as described previously using a tri-spin protocol [70] and re-suspended in 50 μl of analytical grade water. The RNA concentration of samples was estimated using a nanodrop spectrophotometer. The absorbance ratio A_260_:A_280_ was 1.76 ± 0.005 (mean ± S.E.M.) with an average concentration of 43 ng/μl ± 0.5 (mean ± S.E.M.). RNA was diluted to 23–40 ng/μl depending on the experimental set. RNA was primed using random hexamers and reverse transcribed using the superscript II kit (Life Technologies, Paisley, UK) according to manufacturer's instruction.

### 2.3 Taqman Low Density Array (TLDA) analysis

The TLDA (Life Technologies, Paisley, UK) was designed to assess all 23 MMP genes as well as 18 of the 19 ADAMTS genes, all 4 TIMP genes, 10 key proteoglycan and 4 collagen genes, as well as the endogenous control gene 18s. 50 ng (MMP, ADAMTS and TIMP) or 243 ng (proteoglycan and collagens) cDNA was loaded into the fill reservoirs and the plate was run according to manufacturer's instructions (Using the Applied Biosystems 7900HT Real-Time PCR System and Applied Biosystems Sequence Detection Systems (SDS) software [Life Technologies, Paisley, UK]). Undetected samples were given a Ct value of 40. Relative expression levels in each gene of interest were analysed by normalising to endogenous control gene 18s (∆Ct [endogenous control gene Ct–gene of interest Ct]) and expressing the data as 2^∆∆Ct^. All data were normalised to the control, which is represented by the baseline value of 1.

### 2.4 Quantitative Real Time PCR

The standard qRT-PCR programme was run using selected primer probe sets (see Table 1) and the Applied Biosystems 7500 Taqman system. Each reaction was performed in a volume of 25 μl including; 11–19 ng/well of cDNA (depending on the experimental set), 50% KAPA Probe fast qPCR kit Mastermix  (2×) (Anachem, Bedfordshire, UK), 10 nM each of the forward and reverse primer and 5 nM of probe. Standard curves were run for each assay to confirm primer probe efficiency. Relative expression levels in each gene of interest were analysed by normalising to endogenous control genes Topoisomerase-1 (TOP1) and 18s (∆Ct [endogenous control gene Ct–gene of interest Ct]) and expressing the data as 2^∆∆Ct^. All data were normalised to the control, which is represented by the baseline value of 1. Data shown was normalised to TOP1, as GeNorme analysis demonstrated TOP1 to be the most stable housekeeping gene (normalising to 18s yields similar results).

### 2.5 TGFβ luciferase assay

In order to test whether TGFβ protein levels were modified in response to mechanical loading, we measured total and active TGFβ in strain conditioned medium using a cell-based luciferase assay [65]. SW1353 chondrosarcoma cells were transfected with CAGA and Renilla [65] constructs simultaneously (Renilla is used to control for transfection efficiency) using Fugene 6 (Sigma Aldrich, Dorset, UK). Cells were serum-starved for 24 h before 6 h incubation with conditioned medium from 48 h strained/non-strained cultures. Duplicate samples of conditioned medium were heated for 5 min at 80 °C to activate TGFβ before incubation with SW1353 cells. The Promega Dual luciferase™ reporter assay kit (Southampton, UK) was used to measure luciferase activity according to manufacturer's instructions. The absorbance was measured using a PerkinElmer spectrophotometer (Cambridge, UK) at 560 nm. CAGA luciferase units were normalised to Renilla luciferase units to account for transfection efficiency (CAGA Luciferase units/Renilla luciferase units). These values were normalised to the non-conditioned medium controls and expressed as a percentage of the total TGFβ in non-strained controls. Specificity of this assay for TGFβ was determined by the addition of a pan inhibitory antibody against TGFβ or a TGFβRI inhibitor in combination with conditioned medium from multiple strain experiments.

### 2.6 Statistical analysis

Data were presented as a mean ± Standard error (S.E.M.). Data were tested for normal distribution using the Shapiro–Wilk normality test. The Wilcoxon signed rank test and the students paired T test (2 tailed, assuming unequal variance) were used to analyse qRT-PCR data and TGFβ luciferase data using SPSS as appropriate. p < 0.05 was chosen as the cut off for statistical significance.

## 3 Results

### 3.1 Screen of mRNA expression with mechanical strain

**Fig. 1:**
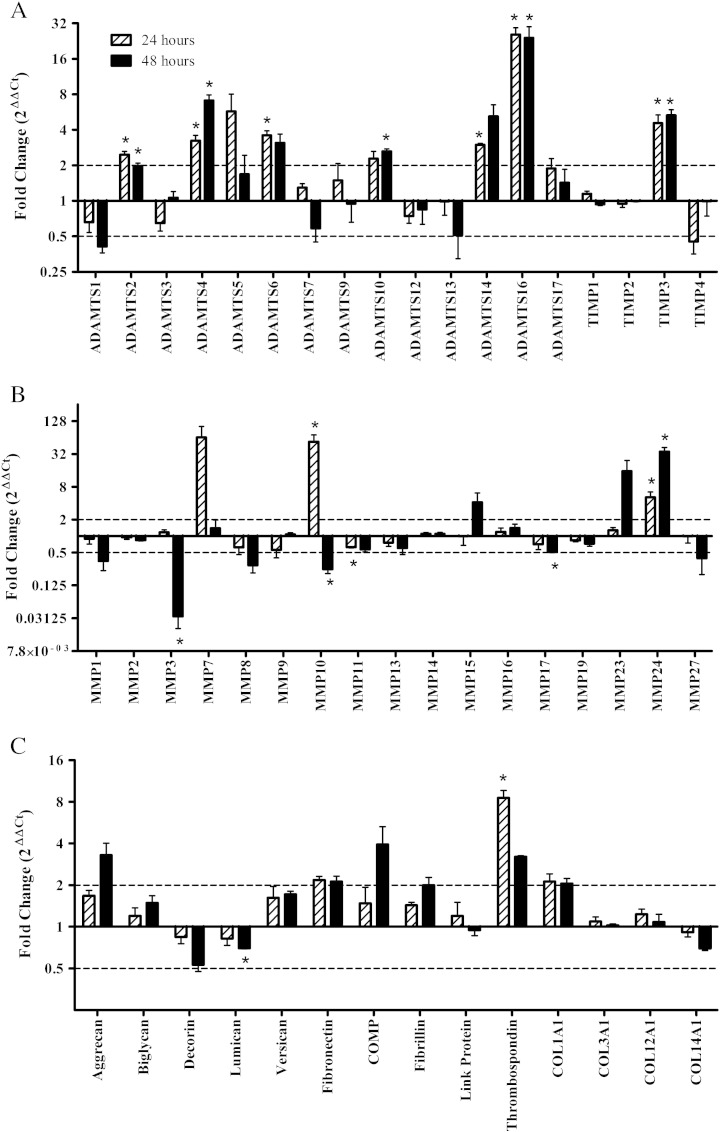
Taqman Low Density Array of strain modulated metalloproteinase and matrix genes; ADAMTS (A), TIMP (A), MMP (B) and matrix proteins (C). Quantitative Real Time PCR (Taqman Low Density Array Data) analysis of cells seeded at 1.5 × 10^6^ cells/ml in type I rat tail collagen at 1 mg/ml following 5% cyclic strain for 24 and 48 h. The dotted line represents the two fold modulation point. Data were normalised to 18s and expressed as a ratio of strain: non-strain (n = 3). Significant values are indicated as * (p < 0.05).

In order to identify strain responsive genes we measured the expression of all 23 MMP, 4 TIMP and 18 ADAMTS mRNA and a selection of matrix genes using a Taqman Low Density Array (Fig. 1). Of these genes we were unable to detect ADAMTS8, ADAMTS15, ADAMTS18, ADAMTS19, MMP12, MMP20, MMP21, MMP25, MMP26, MMP28 and COL2A1.

There was a significant increase in the following genes with strain compared to non-strained controls at 24 and 48 h; ADAMTS2 (2.5 and 2 fold), ADAMTS4 (3.2 and 7.1 fold), ADAMTS16 (25.6 and 24.1 fold), MMP24 (5.2 and 35.2 fold) and TIMP3 (4.6 and 5.3 fold) (at 24 and 48 h respectively). MMP10 was increased with strain after 24 h (53.5 fold) and decreased after 48 h (4 fold). Thrombospondin-1 (8.6 fold), ADAMTS6 (3.6 fold) and ADAMTSS14 (3 fold) were increased and MMP11 was decreased (1.6 fold) after 24 h of strain. Lumican (1.4 fold), MMP3 (33.3 fold) and MMP17 (2 fold) were decreased and ADAMTS10 (2.6 fold) is increased after 48 h of strain.

Using histological analysis we observed that cells became aligned to the axis of load and that there was no difference between strained and non-strained cultures (data not shown), similar to previous reported observations [66].

### 3.2 Screen of mRNA expression with TGFβ

Using a real time PCR array, we confirmed that the majority of gene changes were similar with strain or TGFβ treatment; 12/14 ADAMTSs, 14/17 MMPs and 13/14 of matrix genes showed a similar response to strain and TGFβ at 24 and 48 h. Three out of the four TIMPs also showed similar responses to strain and TGFβ (see Table 2, for a comparison of changes with TGFβ and strain). Genes that showed dissimilar responses included; ADAMTS5, ADAMTS7, MMP7 and MMP16 which were decreased with TGFβ and increased with strain at 24, 24, 48 and 48 h respectively. MMP15 and TIMP4 were increased with TGFβ and decreased with strain at 48 h.

### 3.3 Selected gene regulation with strain or TGFβ

**Fig. 2:**
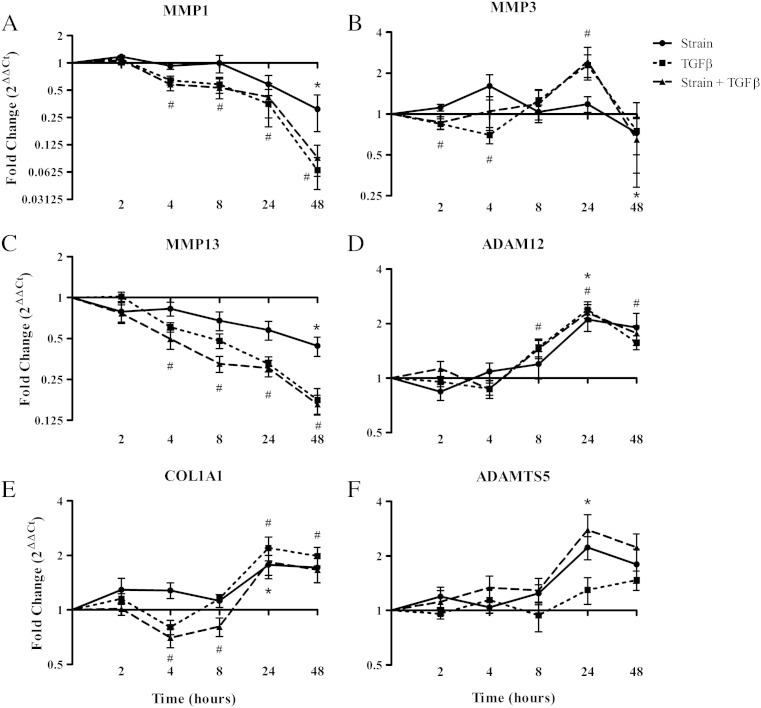
Strain and TGFβ modulation of metalloproteinases and matrix genes. Quantitative Real Time PCR analysis of cells seeded at 1.5 × 10^6^ cells/ml in type I rat tail collagen at 1 mg/ml after 5% cyclic strain over a time course of 0–48 h. Data were normalised to TOP1 and expressed as a ratio of strain: non-strain, TGFβ: control or strain plus TGFβ: control (n > 6). Significant values are indicated as * (changes with strain) or # (changes with TGFβ) (p < 0.05).

We selected metalloproteinase and matrix genes that responded to mechanical strain in the array for further time course analysis. MMP1, MMP2 and MMP13 were selected on the basis of their regulation with strain in the TLDA and key role in tendon matrix turnover. ADAM12 was chosen as we had previously shown its regulation in tendinopathy [36]. COL1A1 was selected as it is the main collagen expressed in tendon and it was regulated with strain in our TLDA screen. ADAMTS5 was chosen as it responded differently to mechanical load and TGFβ, unlike many of the other genes, and it has also been shown to be regulated in tendinopathy. MMP1 (3.3 fold, 15.3 fold) and MMP13 (2.3 fold, 5.7 fold) were significantly decreased with 5% cyclic strain or TGFβ at 48 h (Fig. 2). MMP13 was also significantly decreased (1.7 fold, 3 fold) at 24 h with both strain and TGFβ (error bars are variable due to low expression of MMP13). ADAM12 (2.1 fold, 2.4 fold), and COL1A1 (1.8 fold, 2.2 fold) were significantly increased with 24 h of cyclic strain or TGFβ. ADAMTS5 (2.2 fold) and MMP3 (2.3 fold) were significantly increased at 24 h and MMP3 (1.38 fold) was significantly decreased at 48 h in response to strain. Although ADAMTS5 and MMP3 showed a similar pattern of regulation with TGFβ, this was not significant. The majority of TGFβ mRNA responses in our system show changes analogous to those seen with mechanical strain. Strain responses in the presence of TGFβ show that there is little effect of strain over and above TGFβ stimulation in most cases and in general the responses with TGFβ were more robust than those with strain. However ADAMTS5 was the exception, as TGFβ did not regulate ADAMTS5 significantly.

### 3.4 Inhibition of TGFβRI abrogates the mRNA response to strain

**Fig. 3:**
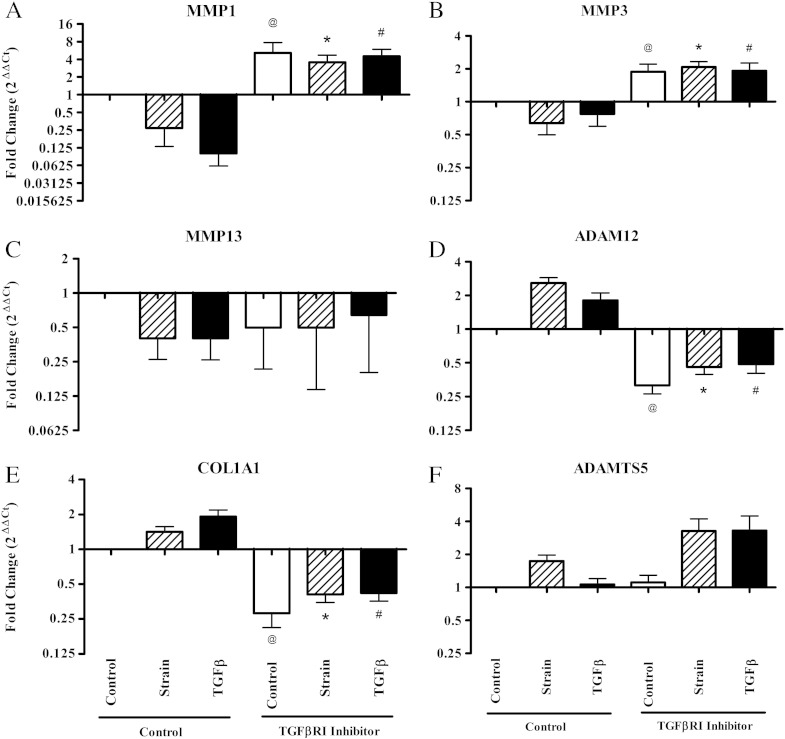
TGFβRI inhibition of strain mediated responses. Quantitative Real Time PCR analysis of cells seeded at 1.5 × 10^6^ cells/ml in type I rat tail collagen at 1 mg/ml (± TGFβRI inhibitor, 10 μM) after 5% cyclic strain at 48 h. Data were normalised to TOP1 and expressed as a ratio of strain: control or TGFβ: control (n > 6). All data were normalised to the control without TGFβRI inhibitor, which is represented by the baseline value 1 in each graph. Significant values are indicated as @ (control compared to control plus inhibitor), * (strain compared to strain plus inhibitor) and # (TGFβ compared to TGFβ plus inhibitor) (p < 0.05).

The addition of a TGFβRI inhibitor abrogated the TGFβ mediated response; In MMP1, MMP3, ADAM12 and COL1A1 mRNA expression there was a significant difference between TGFβ treated cultures ± TGFβRI inhibitor at 48 h (p < 0.05) (Fig. 3). Addition of the TGFβRI inhibitor also abrogated responses to strain in MMP1, MMP3, ADAM12 and COL1A1 at 48 h. This suggests that strain signals via TGFβRI in the TGFβ signalling pathway. Even in the non-strained control cultures the addition of TGFβRI inhibitor caused mRNA levels to change in the opposite direction to the strain response, indicating a basal level of TGFβ signalling in non-strained cultures. This may be due to the tensional forces present across the tenocyte seeded collagen gel in the absence of cyclic loading. ADAMTS5 and MMP13 were an exception, as their regulation appears to be independent of TGFβRI signalling.

### 3.5 Strain regulation of TGFβ protein and mRNA

**Fig. 4:**
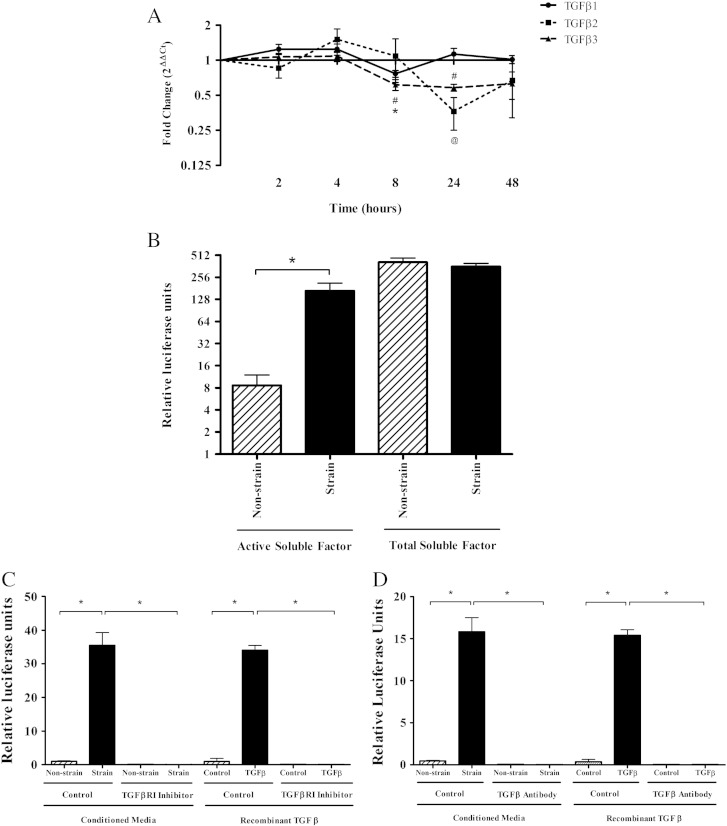
TGFβ activation is increased with mechanical strain. (A) Quantitative Real Time PCR analysis of TGFβ isoforms in cells seeded at 1.5 × 10^6^ cells/ml in type I rat tail collagen at 1 mg/ml after 5% cyclic strain over a time course of 0–48 h. Data were normalised to TOP1 and expressed as a ratio of strain: non-strain (n > 6). Significant values are indicated as * (TGFβ1), @ (TGFβ2) or # (TGFβ3). (B) A cell based luciferase assay measuring levels of SMAD activatory soluble factor (see Methods for details). Data were normalised to transfection controls and negative controls (n = 5). Significant values are indicated as * (p < 0.05). In order to confirm that TGFβ signalling and more specifically TGFβ activity are responsible for the stimulation of SMAD activity, inhibitors of TGFβ activity and TGFβ signalling were used. Non-conditioned media ± TGFβ, strain and non-strain conditioned media were incubated with TGFβRI inhibitor (C) and a Pan specific TGFβ inhibitory antibody (D) to confirm that the measure of SMAD activation was stimulated via TGFβ. Significant values are indicated as * (p < 0.05) (n = 3).

Following our observation that TGFβ signalling plays a role in the response to cyclical strain, we measured TGFβ1, TGFβ2 and TGFβ3 mRNA expression. TGFβ1 and TGFβ3 mRNA were significantly decreased with strain at 8 h (~ 1–2 fold) (Fig. 4A) and TGFβ2 and TGFβ3 mRNA were significantly decreased at 24 h with strain (~ 2 and 3 fold respectively). Therefore there was no significant increase in the level of TGFβ mRNA expressed in response to strain. Therefore, regulation of TGFβ at the mRNA level could not explain the increase in TGFβ signalling stimulated with mechanical strain.

Strain conditioned medium induced a significantly higher level of SMAD activity compared to conditioned medium from non-strained controls (~ 40 fold increase) (Fig. 4B). Heat-activated conditioned medium, measuring total TGFβ, did not show a significant difference in SMAD activity between strained and unstrained cultures. The specificity of this assay was confirmed by the addition of TGFβRI inhibitor and a Pan-TGFβ antibody (inhibits all TGFβ isoforms) to conditioned media and recombinant TGFβ (see Fig. 4C and D). The addition of these inhibitors to strain conditioned media completely abrogated the detection of TGFβ, confirming that the assay is specifically detecting TGFβ. This indicated that activation and not total TGFβ was increased in response to mechanical loading and that TGFβ activation was a key step in the mechanotransduction and regulation of metalloproteinase and matrix genes.

### 3.6 Investigation of TGFβ activation mechanisms

**Fig. 5:**
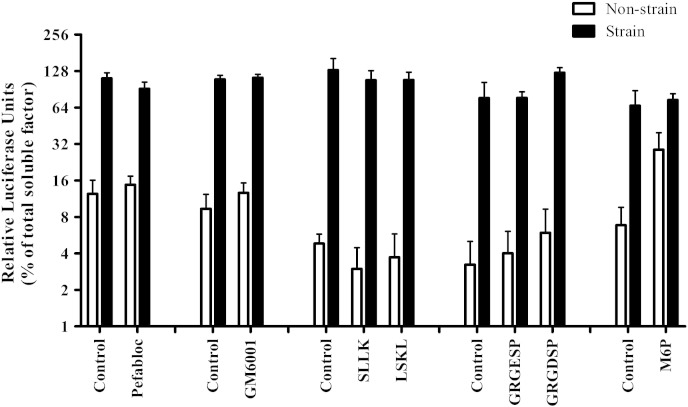
Inhibition of potential TGFβ activation mechanisms. Conditioned media was collected from cells seeded at 1.5 × 10^6^ cells/ml in type I rat tail collagen at 1 mg/ml and cyclically strained at 5% for 48 h with the addition of MMP inhibitor, GM6001 (10 μM); serine protease inhibitor, Pefabloc (0.5 mM); RGD peptide inhibitor, GRGDSP (7 μM); thrombospondin peptide inhibitor, LSKL (13 mM) and Mannose-6-phosphate, M6P (10 μM). A cell based luciferase assay measuring levels of SMAD activatory soluble factor was used to assess TGFβ (see Methods for details). Data were normalised to transfection controls and negative controls before expression as a percentage of total SMAD activatory soluble factor in non-strain samples (n = 3, except the M6P data where n = 1 is shown, this data is representative of 2 other repeats).

MMPs (MMP2, MMP9 and MMP14) and serine proteases (thrombin and plasmin) have been reported to activate TGFβ by cleaving TGFβ from LAP [29,71]. In order to test the involvement of proteases in the activation of TGFβ in response to mechanical strain, a broad spectrum MMP inhibitor (GM6001) and a serine protease inhibitor (pefabloc) were added to tenocyte seeded 3D collagen gels before straining. After 48 h SMAD activity was measured using the luciferase assay. SMAD activity was not significantly different from controls (see Fig. 5). This indicates that the SMAD activatory soluble factor is not activated via protease cleavage; at least those inhibited by GM6001 or pefabloc, in response to mechanical strain.

TGFβ does not require physical separation from the LAP for activation; interaction of LAP with integrin and thrombospondin domains (RGD and LSKL motifs respectively) can result in a conformational change and consequent activation of TGFβ [30,33,67,72]. RGD and LSKL mimetic peptides were added to tenocyte seeded 3D collagen gels before straining to inhibit this interaction. After 48 h strain SMAD activity was measured using the luciferase assay. SMAD activity was not significantly different from controls (see Fig. 5). This indicates that integrin and thrombospondin interaction with latent soluble factor is not involved in strain mediated activation of the SMAD activatory soluble factor.

The M6P/IGF-II receptor has also been reported to be involved in the activation of TGFβ [31,32]. LAP can bind to M6P/IGF-II which causes cell surface localisation of the latent complex and may result in activation of TGFβ through proteolytic cleavage [32]. Addition of Mannose-6-phosphate (M6P) prevents this interaction and therefore inhibits TGFβ activation [32]. M6P was added to tenocyte seeded 3D collagen gels before straining to test whether M6P is involved in strain mediated TGFβ activation. After 48 h strain SMAD activity was measured using the luciferase assay. SMAD activity was not significantly different from controls (see Fig. 5). Therefore none of the well characterised mechanisms of TGFβ activation tested in this study was demonstrably responsible for the strain mediated activation of the SMAD activatory soluble factor. This indicates that a potentially novel mechanism of activation is stimulated in response to mechanical strain in tenocytes.

## 4 Discussion

We are the first to study the effect of mechanical regulation on an array of protease genes in human tenocytes. We have chosen a strain of 5% as it is within the physiological range experienced by human tendons [73,74]; given that some tendons can elongate 12–15% [74], values for cell strain of 4–5% may be considered within the physiological range, at least for highly loaded tendons such as the Achilles. Our data shows that 5% mechanical strain has a potential anabolic effect on the collagenous matrix, as the two main collagenases (MMP1 and MMP13) are decreased with mechanical strain, accompanied by an increase in COL1A1 expression.

There have been a number of studies that have looked at the effects of mechanical strain on MMPs, mainly in rodent tendon in vivo, ex vivo or in vitro cell studies. Obvious limitations of these studies include the differences in species as rodents do not express MMP1; instead they only express MMP13, which is the nearest homologue [47,75]. In some studies 2D models lack the cell–matrix contact that is so obviously important in the 3D matrix seen in vivo [76–78]. Although it is difficult to compare studies of different design, studies focusing on strains of lower magnitude are reported to have an anabolic effect upon the tendon, consistent with the current study [47,48,51,75,79,80]. However, a number of rodent tendon in vivo and in vitro studies have shown no significant change in MMP3 or MMP13 mRNA upon moderate loading [50,51,77,79], this may be due to a lower frequency in strain (< 0.5 Hz) or the reduced loading time. This is consistent with our data as MMP1, MMP3 and MMP13 are not significantly regulated by strain until 48 h.

No other published studies have looked into the mechanical regulation of MMP10, MMP24, the ADAMs, TIMPs 3 and 4 and such a large array of ADAMTS and matrix proteins at either the mRNA or protein level in human tendon. Our data suggests that generally there is a decrease in MMPs (MMP1, -3, -11, -13 and -17) and an increase in collagen at the mRNA level. We have also shown an increase in TIMP3 mRNA which is known to inhibit ADAM12, MMP1, MMP2, MMP3, ADAMTS4 and ADAMTS5 at the protein level [81–83]. Although to confirm this TIMP3 protein quantification and activity measurement are required. This supports earlier reports that moderate cyclic strain is largely anabolic, i.e. maintaining the collagen components of the ECM.

Vogel showed stimulation of aggrecan with TGFβ, which is possibly similar to the current study [84]. The is no evidence to suggest that the tenocytes are differentiating to the chondrogenic phenotype, as chondrogenic markers such as SOX9 and collagen type II are not elevated with mechanical strain and tendon related genes such as tenascin C, thrombospondin 4 and scleraxis are significantly increased with strain. Proteoglycan regulation has also been reported in a rat tail tendon model. After a cyclic loading regime of 3% overlaying a 2% static strain for 24 h decorin, fibronectin and biglycan mRNA was decreased, increased and unchanged respectively [79,85]. The current study showed no significant changes in any of the above proteoglycans. No other published studies have looked into any other proteoglycans at the RNA level in response to mechanical load.

Arnoczky's group has shown that mechanical strain is important in tendon ECM homeostasis in that MMPs are increased with the absence of tension [45,47,48,75]. Data from Smith et al. also support this [86]. In addition, Arnoczky's group has proposed that stress deprivation, caused by isolated tendon fibre damage due to a single abnormal over load, is a key factor in tendinopathy, as opposed to catabolic effects induced by a cellular response to high levels of repetitive strain [87]. Although fatigue loading has been shown to disrupt collagen fibres [88], load induced tendon catabolism requires a level of strain that does not “normally” occur in the tendon in vivo [87]. In the current study we have shown little evidence of load inducing catabolic effects, hence our data support Arnoczky's ideas. Therefore it is important for us to fully elucidate the process of mechanotransduction to truly understand the underlying factors contributing to the development of tendinopathy.

Comparing the responses of metalloproteinase and matrix genes to strain and tendinopathy may shed light on the relationship between mechanical loading and tendinopathy development. MMP2, MMP3, MMP8, ADAMTS2, ADAMTS4, ADAM12 and collagen type I expression were regulated in a similar manner in tendinopathy compared to the current study [15,36,38,57,70,89,90]. However, MMP1, MMP13, MMP10, ADAMTS5, TIMP3, versican, aggrecan, biglycan, decorin, fibronectin, fibrillin and COMP showed an opposite response with strain compared to tendinopathy [10,13,15,36–38,40,89,90]. The similarity between strain responses and changes in tendinopathy in terms of gene expression suggests that an altered strain regime may contribute to the development of tendinopathy. However, variation in response to strain and tendinopathic changes suggests that tendinopathies are complex and there may be other causative factors other than mechanical loading.

TGFβ protein is increased in tendinopathy [18,19]. Pathway analysis following global gene expression profiling of chronic tendinopathic tendon tissue also showed an increase in genes related to TGFβ signalling [89]. We have compared gene regulation in tendinopathy and response to mechanical load. We showed that there were many similarities. However gene expression also suggests that the mechanotransduction mechanism has been disrupted in some way in tendinopathy, as there were also some differences in the response to mechanical load and those seen in tendinopathy. This indicates that TGFβ signalling response to load may be disrupted in some way. Abnormal loading regimes may result in differential response of TGFβ and contribute to the development of tendinopathy.

We have shown that the vast majority of changes reported in response to strain have shown a similar pattern of response to TGFβ. Some of the gene changes in response to TGFβ have previously been reported in other cell types. For example TGFβ is reported to regulate ADAM12 [54], ADAMTS1 [55], ADAMTS4 [56,57], ADAMTS16 [58], TIMP3 [59], TIMP4 [60], thrombospondin [61], aggrecan [62], collagen and fibronectin [63]. These responses are analogous to those seen in our model with TGFβ and strain, further supporting our conclusion that TGFβ plays a key role in the strain regulation of most metalloproteinase genes. ADAMT5 has been shown to either be unresponsive [56] or to decrease [55] in response to TGFβ. This corresponds with the current study in that induction of ADAMTS5 by strain is not related to TGFβ signalling directly. Recent research has also reported that ADAMTS5 can also positively regulate TGFβ signalling through degradation of aggrecan, allowing TGFβ to access receptor molecules [91]. The increase of ADAMTS5 in our system in response to strain may increase TGFβ signalling. Despite the fact that MMP13 shows similar response to strain and TGFβ, regulation does not appear to be mediated via the TGFβRI pathway either. This indicates that regulation of MMP13 by TGFβ is via a non-canonical TGFβ signalling pathway.

In some instances there is an additive effect when both strain and TGFβ are applied. ADAMTS5 is increased significantly with strain after 24 h (2.2 fold), in response to TGFβ there was a trend to increase, however with a combination of strain and TGFβ treatment ADAMTS5 was increased further than strain alone (2.8 fold, 24 h). ADAMTS5 stimulation with strain is not mediated via TGFβRI signalling therefore strain and TGFβ response rely on different mechanisms. We have also noted that the increase in elastin was reduced when strain and TGFβ were combined; elastin was increased 3.8 fold with strain, 27.5 fold with TGFβ and 11.2 fold with strain and TGFβ in combination. We have shown that elastin was regulated via TGFβRI however we have not fully characterised this response. In speculation, this may result from a negative feedback mechanism triggered when the levels of TGFβ reach a threshold; however more research is required to test this hypothesis.

Limitations of the study include the sole use of tendon cells derived from tendinopathic tissue. However, we have studied a number of cell isolates derived from normal and ruptured tendon, which showed similar responses in terms of metalloproteinase regulation with strain (data not shown). This suggests that tendon cells derived from different disease phenotypes respond in a similar way to cells derived from normal tendon when cultured. In addition, as we seed the tenocytes into a simple collagen matrix which is lacking in many of the components of the ECM that occur in vivo, tenocytes may respond differently to mechanical loading than those in native ECM. Further study of TGFβ involvement in gene regulation with mechanical strain in tendon fascicles would confirm whether tenocytes respond in a similar manner in their native environment. Another limitation of this study is that we were unable to confirm that all inhibitors used to characterise mechanisms of TGFβ activation were effective although we were able to confirm the inhibitory ability of GM6001 after 48 h of culture, which was achieved using a quenched fluorescent substrate and active metalloproteinase (data not shown). Other inhibitors were used at concentrations more than or equal to those reported in similar studies (M6P and pefabloc [92]; LSKL [68,69]; RGD peptide [67]).

Taken together our data suggest that activation of TGFβ and subsequent TGFβ signalling is part of the mechanotransduction response of tenocytes to moderately high levels of strain. TGFβ up-regulation at both the protein and mRNA level with mechanical strain has been previously reported in tenocytes [93,94] and other cell types [95–98]. TGFβ has also been implicated as a regulatory step in the mechanical regulation of collagen [94,99], although these studies have not specifically shown that activation of TGFβ is involved. Maeda et al. [100] have reported TGFβ activation as a key regulator of scleraxis expression (a transcription factor expressed in the developing tendon rudiment); although they did not how total levels of TGFβ. We are the first to implicate TGFβ activation (and not protein or mRNA synthesis) as a key regulator in the mechanoregulation of metalloproteinases and other matrix genes. As ADAMTS5 is not regulated via the same TGFβ signalling pathway, this suggests that there is an alternative mechanotransduction pathway. This pathway may involve crosstalk with or may precede TGFβ activation. The next goal is to elucidate the mechanism by which mechanical strain induces latent TGFβ activation, since doing so could help us to fully understand how we could treat and ultimately prevent tendon disease.

## 5 Conclusions

We have shown that mechanical strain regulates multiple protease and matrix genes at the mRNA level and that changes in mRNA level are analogous to those induced by TGFβ stimulation. Furthermore, the inhibition of the TGFβ signalling pathway abrogated the strain-induced changes in mRNA level, demonstrating signalling via TGFβRI which mediates downstream phosphorylation of SMAD [64]. This indicates that signalling is mediated via the canonical TGFβ signalling pathway which involves SMAD translocation to the nucleus and consequent transcriptional regulation. In further support of this we have shown that activation of TGFβ is increased in response to mechanical load and that activation rather than synthesis of TGFβ is important in the mechanical regulation we observed. We therefore hypothesise that application of 5% strain at 1 Hz in our model induces TGFβ activation and subsequent signalling.

## Figures and Tables

**Table 1:** Quantitative Real Time PCR primer probe sets.

Gene	Sequences	Reference
MMP1	Forward primer: 5′-AAGATGAAAGGTGGACCAACAATT-3′ Reverse primer: 5′-CCAAGAGAATGGCCGAGTTC-3′ Probe: 5′-FAM-CAGAGAGTACAACTTACATCGTGTTGCGGCTC-TAMRA-3′	[101]
MMP3	Forward primer: 5′-TTCCGCCTGTCTCAAGATGATAT-3′ Reverse primer: 5′-AAAGGACAAAGCAGGATCACAGTT-3′ Probe: 5′-FAM-TCAGTCCCTCTATGGACCTCCCCCTGAC-TAMRA-3′	[102]
MMP13	Forward primer: 5′-AAATTATGGAGGAGATGCCCATT-3′ Reverse primer: 5′-TCCTTGGAGTGGTCAAGACCTAA-3′ Probe: 5′-FAM-CTACAACTTGTTTCTTGTTGCTGCGCATGA-TAMRA-3′	[102]
ADAM12	Forward primer: 5′-AGCTATGTCTTAGAACCAATGAAAAGTG-3′ Reverse primer: 5′-CCCCGGACGCTTTTCAG-3′ Probe: 5′-FAM-ACCAACAGATACAAACTCTTCCCAGCGAAGA-TAMRA-3′	[36]
COL1A1	Forward primer: 5′-CTGGTCACCATGGTGATCAAG-3′ Reverse primer: 5′-GCAGGCGGGAGGACTTG-3′ Probe: 5′-CTGTCGATGGCTGCACGAGTCACAC-TAMRA-3′	[70]
ADAMTS5	Forward primer: 5′-TGTCCTGCCAGCGGATGT-3′ Reverse primer: 5′-ACGGAATTACTGTACGGCCTACA-3′ Probe: 5′-FAM-TTCTCCAAAGGTGACCGATGGCACTG-TAMRA-3′	[103]
TGFβ1	Forward primer: 5′-TGAGGGCTTTCGCCTTAGC-3′ Reverse primer: 5′-CGGTAGTGAACCCGTTGATGT-3′ Probe: 5′-FAM-CTCCTGTGACAGCAGGGATAACACACTGC-TAMRA-3′	Own design
TGFβ2	Forward primer: 5′-ACGGATTGAGCTATATCAGATTCTCA-3′ Reverse primer: 5′-AACAGCATCAGTTACATCGAAGGA-3′ Probe: 5′-FAM-TTTAACATCTCCAACCCAGCGCTACATCG-TAMRA-3′	Own design
TGFβ3	Forward primer: 5′-TGTCACACCTTTCAGCCCAAT-3′ Reverse primer: 5′-CTCCACGGCCATGGTCAT-3′ Probe: 5′-FAM-ATTGTCCACGCCTTTGAATTTGATTTCCAT-TAMRA-3′	Own design
TOP1	Primer design kit	Primer design kit
18s	Forward primer: 5′-GCCGCTAGAGGTGAAATTCTTG-3′ Reverse primer: 5′-CATTCTTGGCAAATGCTTTCG-3′ Probe: 5′-ACCGGCGCAAGACGGACCAG-3′	[13]

**Table 2:** Comparison of changes in gene expression with strain and TGFβ. Fold changes with strain and TGFβ were calculated compared to controls (non-strained and non-treated with TGFβ). Increases in gene expression are represented by +, decreases in gene expression are represented by −.

	24 h		48 h	
	TGFβ	Strain	TGFβ	Strain
ADAMTS1	−	−	−	−
ADAMTS2	−	−	−	−
ADAMTS3	−	−	−	−
ADAMTS4	+	+	+	+
ADAMTS5	−	+	−	−
ADAMTS6	+	+	+	+
ADAMTS7	−	−	−	−
ADAMTS9	−	−	−	−
ADAMTS10	−	−	−	+
ADAMTS12	−	−	−	−
ADAMTS13	−	−	−	−
ADAMTS14	+	+	−	+
ADAMTS16	+	+	+	+
ADAMTS17	−	−	−	−
MMP1	−	−	−	−
MMP2	−	−	−	−
MMP3	+	−	−	−
MMP7	+	+	−	−
MMP8	−	−	−	−
MMP9	−	−	−	−
MMP10	+	+	−	−
MMP11	−	−	−	−
MMP13	−	−	−	−
MMP14	−	−	−	−
MMP15	+	−		
MMP16	−	−	−	+
MMP17	−	−	−	−
MMP19	−	−	−	−
MMP23	+	−	−	−
MMP24	+	+	+	+
MMP27	−	−	+	+
TIMP1	−	−	−	−
TIMP2	−	−	−	−
TIMP3	+	+	−	−
TIMP4	−	−	−	−
Aggrecan	−	−	+	+
Biglycan	−	−	−	−
Decorin	−	−	−	−
Lumican	−	−	−	−
Versican	−	−	−	−
Fibronectin	−	−	−	−
COMP	−	−	+	+
Fibrillin	+	+	+	+
Link protein	−	−	−	−
Thrombospondin	+	+	−	+
COL1A1	+	+	+	+
COL3A1	−	−	−	−
COL12A1	−	−	−	−
COL14A1	−	−	−	−
