# Corrigendum to “Cyclical strain modulates metalloprotease and matrix gene expression in human tenocytes via activation of TGFβ” [Biochim. Biophys. Acta (2013) 2596–2607]

**DOI:** 10.1016/j.bbamcr.2013.08.007

**Published:** 2013-12

**Authors:** Eleanor R. Jones, Gavin C. Jones, Kirsten Legerlotz, Graham P. Riley

**Affiliations:** Soft Tissue Research Group, School of Biological Sciences, University of East Anglia, Norwich Research Park, Norwich, UK

The authors regret that Table 2 in the article referenced above has a few errors. The corrected table is listed below.

**Table 2:** Comparison of changes in gene expression with Strain and TGFβ. Fold changes with strain and TGFβ were calculated compared to controls (non-strained and non treated with TGFβ). Increases in gene expression are represented by +, decreases in gene expression are represented by –.

	24 h		48 h	
	TGFβ	Strain	TGFβ	Strain
ADAMTS1	−	−	−	−
ADAMTS2	+	+	+	+
ADAMTS3	−	−	+	+
ADAMTS4	+	+	+	+
ADAMTS5	−	+	+	+
ADAMTS6	+	+	+	+
ADAMTS7	−	+	−	−
ADAMTS9	+	+	−	−
ADAMTS10	+	+	+	+
ADAMTS12	−	−	−	−
ADAMTS13	−	−	−	−
ADAMTS14	+	+	+	+
ADAMTS16	+	+	+	+
ADAMTS17	+	+	+	+
MMP1	−	−	−	−
MMP2	−	−	−	−
MMP3	+	+	−	−
MMP7	+	+	−	+
MMP8	−	−	−	−
MMP9	−	−	+	+
MMP10	+	+	−	−
MMP11	−	−	−	−
MMP13	−	−	−	−
MMP14	−	+	−	+
MMP15	+	−	+	+
MMP16	+	+	−	+
MMP17	−	−	−	−
MMP19	−	−	−	−
MMP23	+	+	+	+
MMP24	+	+	+	+
MMP27	−	−	−	−
TIMP1	+	+	−	−
TIMP2	−	−	−	−
TIMP3	+	+	+	+
TIMP4	+	−	+	−
Aggrecan	+	+	+	+
Biglycan	+	+	+	+
Decorin	−	−	−	−
Lumican	−	−	−	−
Versican	+	+	+	+
Fibronectin	+	+	+	+
COMP	+	+	+	+
Fibrillin	+	+	+	+
Link protein	+	+	−	−
Thrombospondin	+	+	−	+
COL1A1	+	+	+	+
COL3A1	+	+	+	+
COL12A1	−	+	−	+
COL14A1	−	−	−	−

The authors would like to apologise for any inconvenience caused.

